# Can we tell where our bodies end and the external world begins? Evidence for precise three-dimensional internal body models

**DOI:** 10.1098/rspb.2025.1255

**Published:** 2025-08-13

**Authors:** Celia R. Blaise, Holly C. Clark, Hannes P. Saal

**Affiliations:** ^1^ Active Touch Laboratory, School of Psychology, University of Sheffield, Sheffield S1 4DP, UK; ^2^ School of History, Philosophy and Digital Humanities, University of Sheffield, Sheffield S1 4DT, UK; ^3^ Insigneo Institute for in silico Medicine, University of Sheffield, Sheffield S1 3JD, UK

**Keywords:** spatial cognition, psychophysics, body representation

## Abstract

Distinguishing our body from the external world is crucial for self-perception and environmental interaction. Yet, the accuracy with which we perceive this boundary remains underexplored. Here, we developed a psychophysical protocol to assess how accurately individuals perceive their body boundaries. Participants were asked whether the midpoint between two tactile stimuli was inside or outside their perceived body boundary. Three-dimensional scans provided objective anatomical boundaries, allowing psychometric functions to be fitted. Results revealed remarkable overall precision, often within millimetres, in localizing body boundaries across multiple body regions. However, accuracy varied: while palm boundaries were localized nearly perfectly, stimuli along the wrist boundaries were frequently misjudged as extending beyond their true anatomical limit, revealing a systematic perceptual bias. Perceptual judgements adapted to changes in posture, but accuracy declined when the detailed local three-dimensional structure was omitted, indicating that proprioceptive cues are combined with detailed local body models. Finally, participants whose anatomy deviated from the average tended to align their responses with a typical body model rather than their unique physiology, suggesting that top-down processes influence boundary judgements. Our findings suggest that body boundary representation combines detailed three-dimensional body models with proprioceptive feedback into an integrated perceptual model of the anatomical body.

## 1. Introduction

Our sense of where our body ends and the external world begins can fluctuate dramatically. At times, this boundary can feel sharp and distinct, such as when plunging into a cold swimming pool. In other moments, it appears fuzzy or indistinct: reclining on a comfortable couch can blur the sense of where one’s body ends and the couch begins ([1, p. 97]; see also discussion in [2]). Importantly, our perception of this boundary does not necessarily align with our body’s actual physical borders [3]. While the latter are defined by their anatomical structure, the former can fluctuate widely, ranging from feeling strongly separated from the environment to experiencing an almost seamless continuity with it [3–5]. To date, body boundaries have primarily been investigated in terms of their experienced sharpness, whereas the accuracy with which this boundary can be consciously located in space remains poorly understood. Here, we focus on body boundaries as the perceptual edge of the anatomical body; that is, the precise location of where our physical body ends.

Perceiving the boundaries of our body likely relies on underlying body representations, which broadly refers to the perceptual, cognitive, sensorimotor and affective processes that shape our experience of bodily form, position, movement and ownership [6]. While it is widely acknowledged that multiple interacting systems (e.g. body schema, body image) contribute to this representation, there is still active debate regarding the precise number and specific functions of these components [7–9]. At the very least, recent frameworks distinguish between two levels of body representation: somatoperception and somatorepresentation [10]. Somatoperception refers to real-time, sensory-driven processes, such as tactile localization, proprioceptive tracking and immediate judgements of body dimensions, whereas somatorepresentation encompasses more stable, higher-order beliefs about one’s body, such as semantic knowledge of body-part arrangement.

So far, research has primarily focused on coarse, large-scale representations (e.g. entire limbs). These studies have consistently revealed substantial and systematic distortions in body representations (see [11] for a comprehensive review). For instance, investigations into perceived body part size have demonstrated significant inaccuracies in judging their relative dimensions [12,13], volume [14] or when localizing landmarks [15–17]. Although these studies have significantly advanced our understanding of body representation, the precision with which fine-grained local body geometry is represented remains largely unexplored. Research on tactile distance perception (e.g. [18]) has partially addressed this gap, revealing smaller scale perceptual distortions that reflect biases in the cortical somatosensory homuncular map [19]. However, these investigations typically consider body parts as mostly flat, two-dimensional surfaces, neglecting its inherently three-dimensional structure. Even seemingly flat regions, such as a hand resting on a table, have pronounced depth and curvature and exhibit intricate three-dimensional geometric structures.

Here, we address this gap by examining whether people can accurately judge their own three-dimensional body boundaries at a fine-grained scale across five individual experiments. First, we compare boundary perception in two body regions with contrasting sensory profiles: the hand; a highly visible, sensitive and frequently used body part, versus the ankle, which is typically out of sight and less sensitive. Next, we investigate how changing hand posture might influence boundary judgements, thereby testing whether the underlying representations are sufficiently flexible to adapt to shifts in proprioceptive feedback. We then ask whether a detailed three-dimensional body model is required to explain people’s performance or whether coarser representations suffice. Finally, in an exploratory analysis, we investigate whether participants whose body deviates from the average shape make judgements aligned with their own body geometry.

## 2. Results

**Figure 1.**
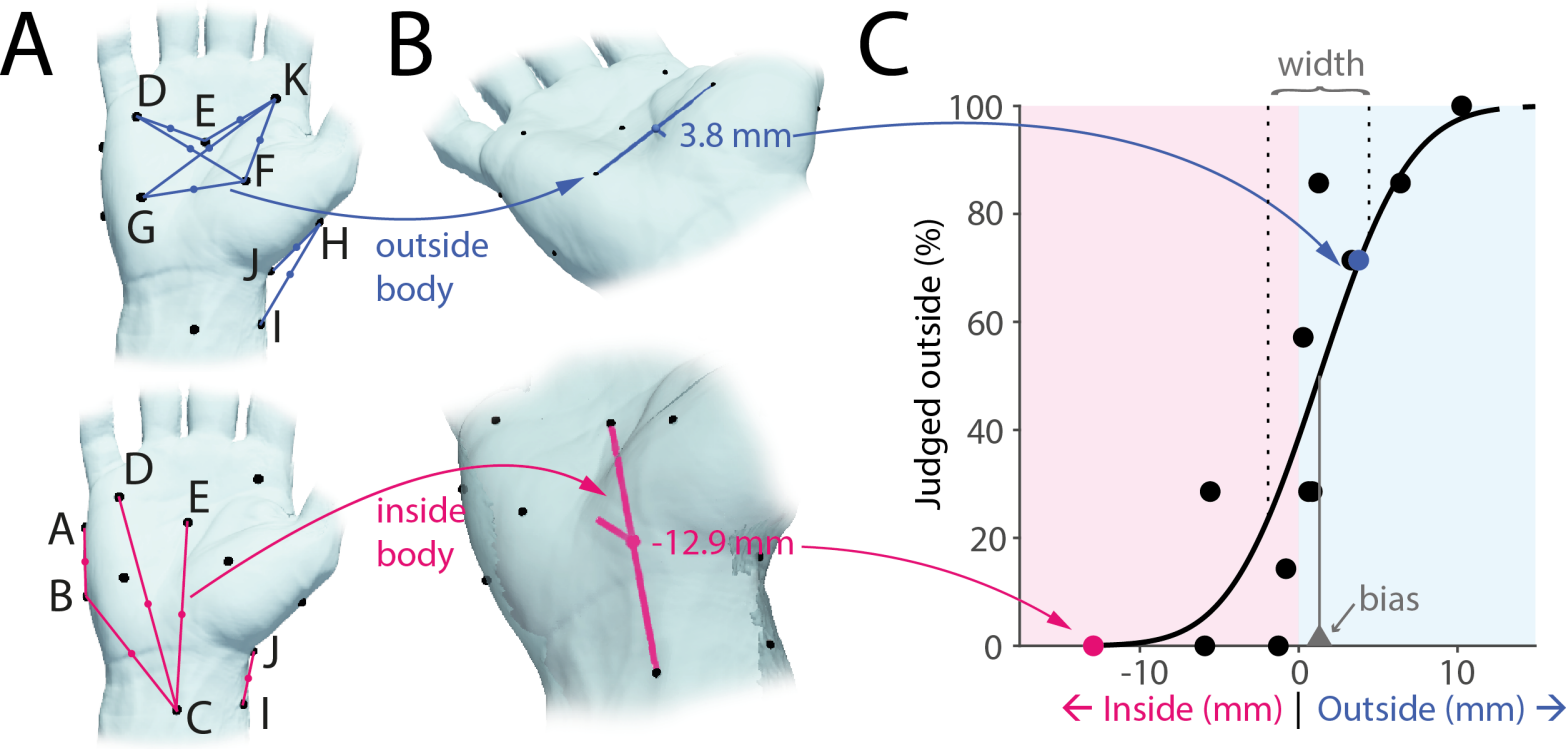
Experimental paradigm. (A) Participants were touched simultaneously at two points (black dots) and asked to judge whether the midpoint of the imaginary line connecting both touches (coloured lines and dots) was perceived to lie inside or outside of their body. Top: midpoints objectively outside the body for a single participant shown in blue. Bottom: midpoints inside the body for the same participant shown in pink. (B) Three-dimensional scanning was used to establish the true distance of each midpoint from the body boundary (two examples shown). (C) A psychophysical curve (black line) was fitted to the perceptual judgements (dots) allowing quantification of perceptual bias (vertical line at 50% perceptual threshold) and width of the body boundary (space between vertical dashed lines at 25% and 75%, respectively).

We developed a novel psychophysical paradigm for assessing the accuracy of the perception of the body boundary. We were specifically interested in quantifying the extent to which the local three-dimensional geometry of different body parts is perceptually accessible in the absence of vision. Participants received simultaneous touches at two predefined points on a given body part and judged whether the midpoint between these points fell within or outside their body. For instance, the midpoint connecting two points G and F might objectively fall outside the boundary of the hand (figure 1A,B, top), while the midpoint connecting points C and E might fall inside (figure 1A,B, pink). Across experiments, we defined 13 such point pairs spanning inside, outside and near-boundary locations. To account for individual anatomical differences, we recorded three-dimensional scans of each participant’s body part, then calculated the actual distance of each midpoint from the true boundary. Finally, combining these distances with each participant’s responses (across seven randomized trials per midpoint) enabled us to fit psychophysical curves and estimate both bias and boundary width (figure 1C).

### (a) Millimetre precision of body boundary perception on the hand

**Figure 2.**
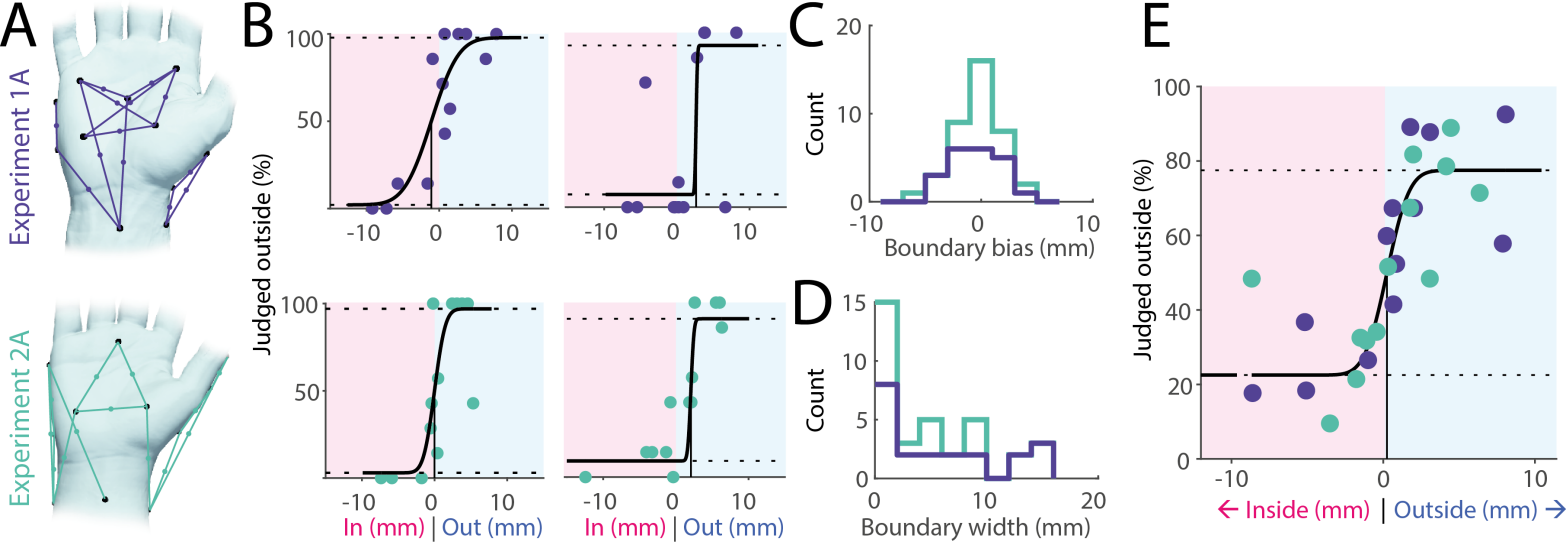
Body boundary perception on the hand. (A) Touch point pairs and their corresponding midpoints tested in experiment 1A (*n* = 21, top) and experiment 2A (*n* = 18, bottom) illustrated for a single participant each. In both experiments, the hand was in a relaxed position, resting relatively flat on a table. (B) Examples of individual fitted psychophysical curves from both experiments. (C) Perceptual bias (point of subjective equivalence) across all participants. Bias was close to zero and the subjective body boundary was as likely to fall slightly outside the true boundary as it was to fall inside. (D) Perceptual width of body boundary as assessed by the distance between judging midpoints as mostly inside (25% threshold) and outside (75% threshold). Almost half of the participants showed precision within a single millimetre and almost all within 1 cm. (E) Group-level psychophysical curve across both studies averaging across individual midpoint distances and responses across 26 unique midpoints.

We first examined participants’ ability to locate their right hand’s boundary while it rested in a relaxed, relatively flat position on a table. Across two separate experiments, each using a different set of midpoints (see figure 2A) and including 39 participants in total, our results indicate that most participants (37 out of 39, 95%) judged the two most outside points more often as falling outside than the two most inside points, with the difference on average 50%, and >25% for 77% of participants, and thus our task generally spanned the range of perceivable distances. Indeed, many of the participants were remarkably accurate as demonstrated by steep, unbiased and well-fitted psychophysical curves (see figure 2B for examples).

Across all participants, the bias in boundary perception (assessed as the point of subjective equivalence) was on average −0.4 mm (see figure 2C for a histogram), not significantly different from zero (*t*(38) = −1.17, *p* = 0.25, *d* = −0.18, one-sample *t*‐test) and did not differ between the two studies (*t*(37) = −1.1, *p* = 0.28, *d* = −0.34, two-sample *t*‐test). Thus, there is no inherent bias to perceive the body boundary as either concave or convex. To assess the width of the body boundary, we calculated the change in distance necessary to jump from a 25% likelihood that a stimulus was judged as outside to a 75% likelihood. On average, the boundary width was 4.9 mm, though for almost half of the participants, it was less than 2 mm (figure 2D). Thus, decisions on whether individual points in space lie inside or outside of the body are made highly precisely within a few millimetres and often within a single millimetre.

Lastly, by combining data from both experiments and fitting a single psychophysical curve at the group level (figure 2E), we again confirmed millimetre-level precision without systematic bias. Nonetheless, some individual points clearly stood out as consistently misclassified by multiple participants, despite their anatomical position being unambiguously inside or outside the body (see examples in figure 2B, top right and bottom left). These recurring errors highlight localized perceptual anomalies rather than random judgement mistakes.

### (b) High fidelity of body boundary perception on the ankle

**Figure 3.**
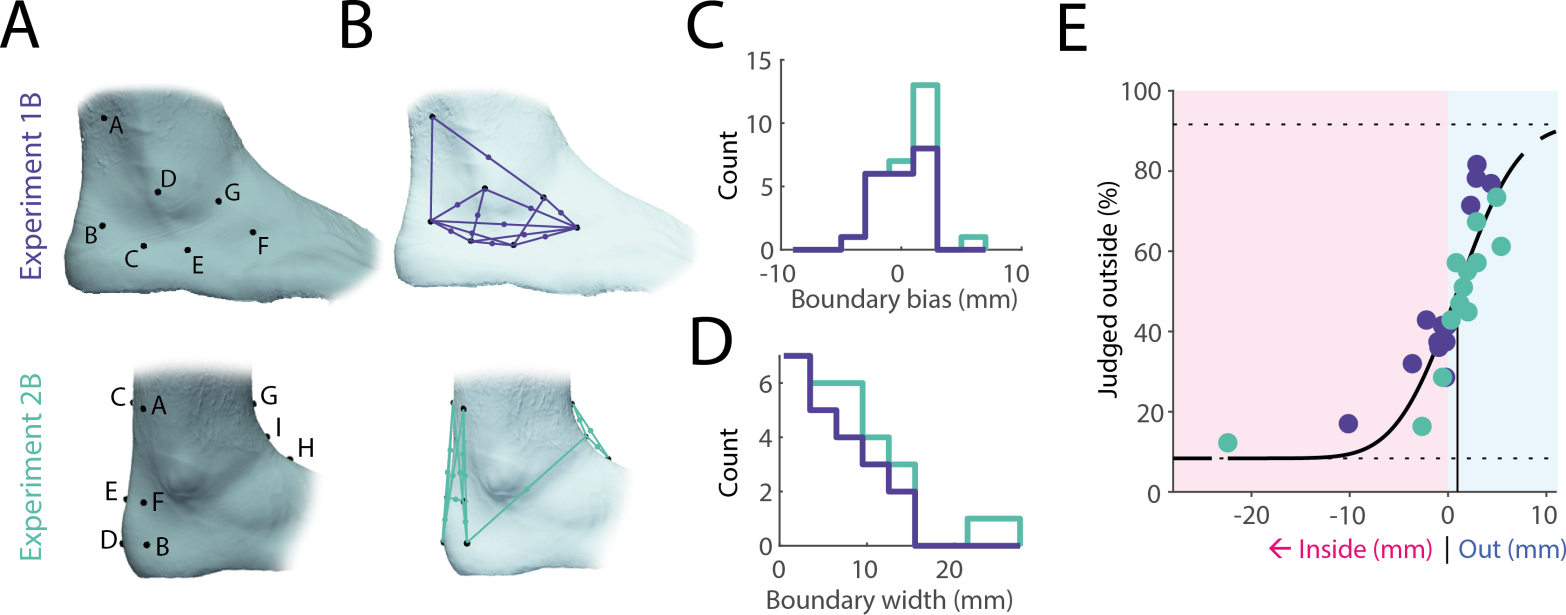
Body boundary perception at the ankle. (A) Touch points on the ankle for experiment 1B (*n* = 21) and experiment 2B (*n* = 7). (B) Corresponding touch point pairs and respective midpoints, focusing on the local geometry around the lateral malleolus for experiment 1B, and the anterior and posterior foot geometry around the ankle for experiment 2B. (C) Bias across all participants. Analogous to the hand, bias was close to zero. (D) Boundary width on the ankle for all participants (see figure 2D for comparison with the hand). Most participants showed precision within 1 cm, slightly less precise than on the hand. (E) Group-level psychophysical curve across both studies averaging across individual midpoint distances and responses across 26 unique midpoints.

Next, we tested whether body boundary perception would differ on another body part, the ankle. Across two experiments (1B: *n* = 21; 2B: *n* = 7), we collected perceptual judgements across 13 different paired touches, following the same procedure established for the hand (see figure 3A for touch points and figure 3B for pairs and respective midpoints). The average bias in boundary perception was 0.6 mm (see figure 3C for histogram), not significantly different from zero (*t*(27) = 1.66, *p* = 0.11, *d* = 0.31, one-sample *t*‐test) or from the bias for the hand (*t*(65) = 1.95, *p* = 0.06, *d* = 0.48, two-sample *t*‐test). The boundary width, at an average of 7.3 mm (figure 3D), was larger than for the hand, though this difference was also not significant (*t*(61) = 1.68, *p* = 0.10, *d* = 0.42). These trends were confirmed in the group-level psychophysical curve (figure 3E), which displayed no bias and high precision across 26 midpoints. In contrast to the results from the hand, no clear outliers were present on the ankle.

### (c) Boundary perception adjusts with changes in posture

**Figure 4.**
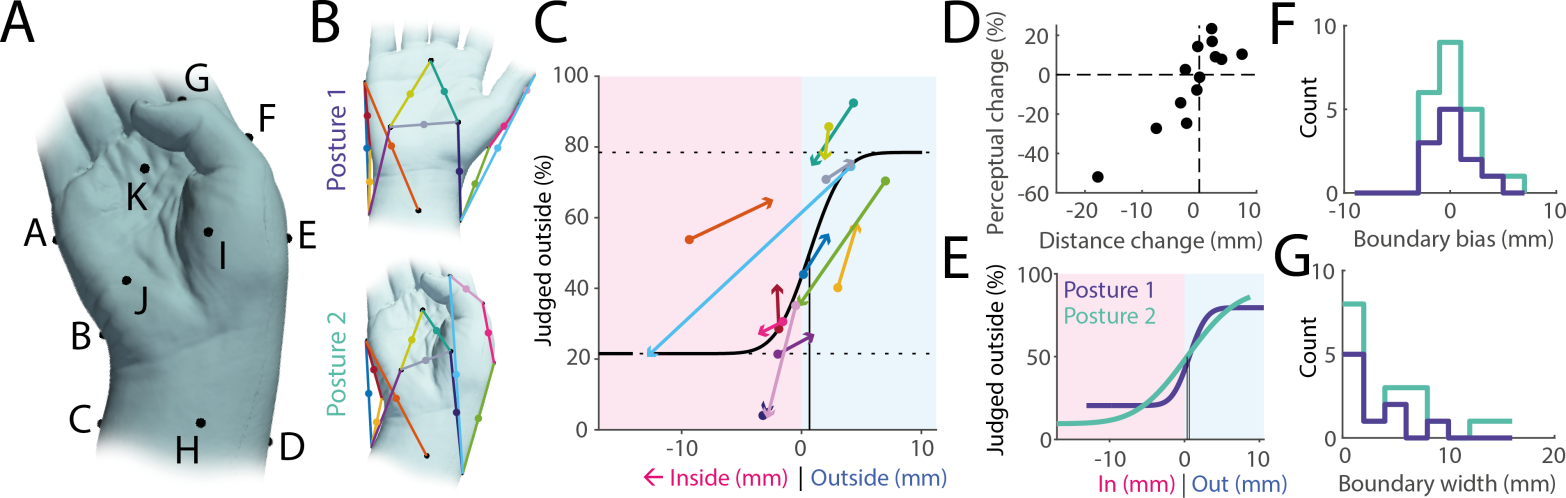
Boundary perception adjusts with changes in posture. (A) Hand posture adopted in experiment 2C, characterized by ulnar deviation and partial flexion of the thumb. (B) Posture 2 was chosen such that, compared with the original baseline posture 1, many midpoints moved with respect to the body boundary, transitioning from inside to outside and *vice versa*. (C) Group-level psychophysical curve (black line) along with boundary distances and perceptual judgements averaged over participants: each coloured line denotes a tested midpoint (same colours as in panel B), with round symbols denoting data from posture 1 and arrow heads denoting data from posture 2. As midpoints move from inside the body to outside, perceptual judgements do the same, and *vice versa*. (D) Change in distance of midpoints from the body surface between postures 1 and 2 compared with accompanying perceptual change, averaged over all participants. Positive distance change values indicate points moving towards the outside of the body, while negative values denote points moving towards the inside. Positive perceptual change values indicate a higher percentage of stimuli being judged as outside, and *vice versa*. (E) Group-level psychophysical curves for posture 1 (dark blue) and posture 2 (green). Their bias is identical and close to zero. The curve is slightly steeper for posture 1. (F) Individual estimates for bias in boundary perception for both postures. Bias is, on average, close to zero and does not differ between postures. (G) Boundary width for all participants across both postures. Precision does not differ between postures.

To determine whether hand posture is taken into account when making perceptual body boundary judgements, we tested the same 13 midpoints under two different postures in a subset of participants (*n* = 11): the baseline relaxed posture and a more ‘unnatural’ posture involving ulnar deviation at the wrist and partial flexion of the thumb (figure 4A). This second posture induced shifts in the tested midpoints, such that some moved from inside the body to outside and *vice versa*: midpoints on the ulnar side of the hand moved towards the outside due to ulnar deviation, while midpoints on the hand’s radial side tended to move inside due to flexion of the thumb (figure 4B). We found that participants’ perceptual judgements shifted in parallel, matching these positional changes in 11 out of 13 midpoints (figure 4C), and the magnitude of perceptual shift was strongly correlated with the physical shift (*r* = 0.87, *p* < 0.01, figure 4D).

At the group level, the psychophysical curves for both postures were highly similar (figure 4E). Although the slope was slightly shallower for the second posture, the difference was not statistically significant due to overlapping confidence intervals and no difference in bias. Individual-level analyses also showed no significant differences in bias (*t*(10) = −0.40, *p* = 0.70, *d* = −0.11, paired *t*‐test, figure 4F) or boundary width (*t*(10) = −1.91, *p* = 0.08, *d* = −0.88, paired *t*‐test, figure 4G) between postures, though given the relatively small number of participants in this task this analysis might have missed smaller effects. We conclude that body boundary perception reflects changes to posture, maintaining broadly similar levels of accuracy.

### (d) Boundary perception reflects local three-dimensional body geometry

**Figure 5.**
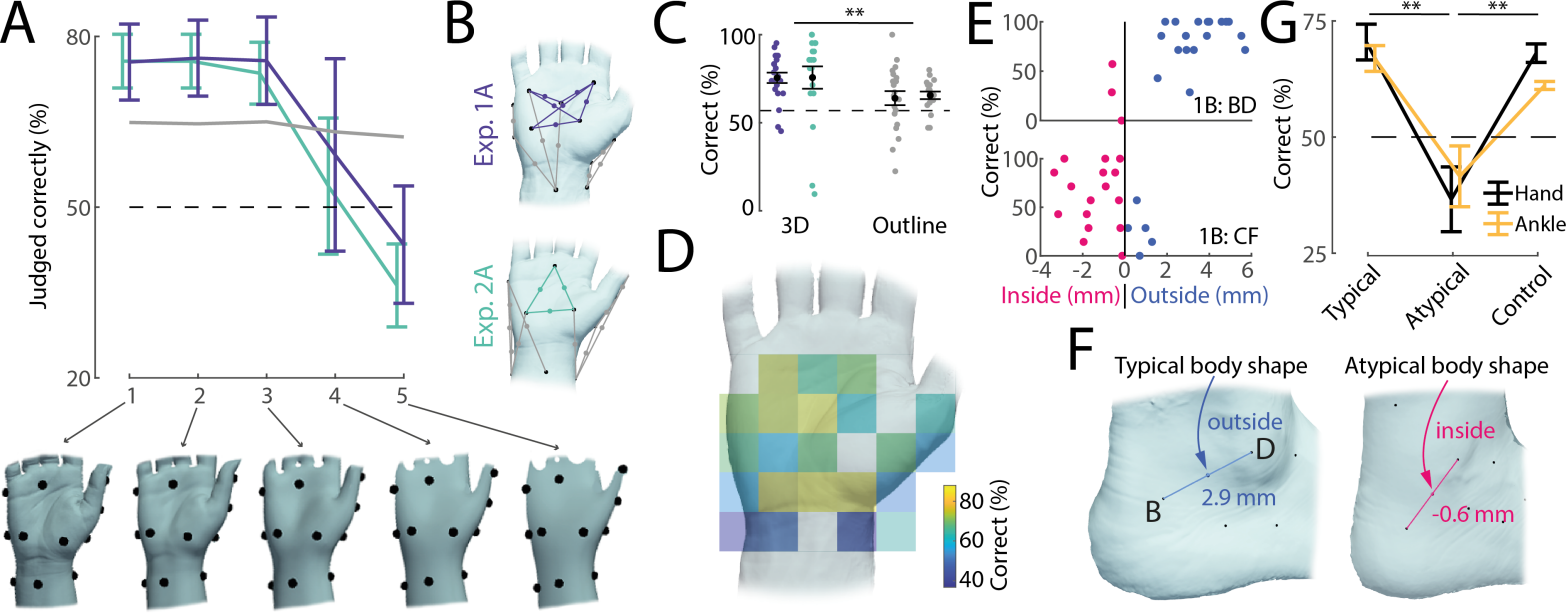
Boundary perception reflects local, typical geometry of the body. (A) Accuracy of perceptual judgements when recalculated with respect to progressively smoothed models of the hand (1: measured high-resolution model, 5: heavily smoothed), shown below. Coloured traces refer to perceptual judgements related to local three-dimensional structure of the hand (as shown in panel B), with lines reflecting averages and error bars denoting standard error of the mean across different midpoints. The grey line is the average accuracy for all other perceptual judgements, mostly reflecting the two-dimensional outline of the hand (see panel B). The dashed line indicates chance level. Accuracy drops considerably for models 4 and 5. (B) Midpoints included in smoothing analysis shown in panel A using the same colour scheme. (C) Accuracy of perceptual judgements reflecting local three-dimensional structure (left) versus two-dimensional hand outlines (right). Black markers denote the average and standard error of the mean across participants, with individual data points shown as coloured dots (same colour scheme as in panel B). (D) Accuracy of perceptual judgements when plotted spatially according to the location of the judged midpoint. Each judgement from every participant (*n* = 39) in the hand baseline posture (experiments 1A and 2A) was first mapped onto the reference hand shown using procrustes analysis, after which averages were calculated for each spatial bin. Accuracy is markedly lower just below the wrist and on both sides of the hand, but high on the palm. (E) Examples of midpoints on the ankle (experiment 1B) falling on different sides of the body boundary for different participants and their associated perceptual judgements. Shown is distance to the actual body boundary on the horizontal axis against the percentage of correct answers. Each point shown is a different participant. Participants whose body boundary does not agree with the typical body shape display low accuracy. (F) Examples of a typical (left) and atypical (right) body shape on the ankle (taken from experiment 1B). Typically, the lateral malleolus is pronounced enough to cause the midpoint between B and D to fall outside the body boundary. However, for a minority of participants, this body region is flattened instead, leading for the midpoint to fall inside the body. (G) Average accuracy for hand (black lines, experiments 1A and 2A) and ankle (yellow, experiments 1B and 2B) judgements where the participant’s body shape agreed with the average body shape (‘typical’, left), where the participant’s body outline disagreed with the average body shape (‘atypical’, middle) and all other judgements made by the same participants included in the atypical dataset (‘control’, right). Participants with atypical body shape show much lower accuracy on these judgements than participants with a typical body shape, even though other judgements by these participants do not display lower accuracy.

We next investigated whether participants’ judgements depended on detailed three-dimensional body representations or on simpler, two-dimensional outlines influenced by posture alone. To do this, we progressively smoothed each three-dimensional hand model (figure 5A, bottom row) and then compared participants’ judgements with the new, smoothed boundaries (see §4). If people rely on fine-grained local geometry, removing these details should reduce accuracy. Conversely, if the mental representation is relatively coarse, smoothing might not affect performance; or could even improve it.

Since smoothing does not change the broad outline of the hand, but instead affects local geometry only, we divided all midpoints tested in the hand baseline condition across both experiments into two categories: those reflecting predominantly local three-dimensional geometry (see coloured markers in figure 5B, located on the palm) and those reflecting simple two-dimensional body outlines or the coarse postural configuration of the hand (grey markers in figure 5B). For example, the palm area exhibits local topography even when the hand is resting flat on the table and correct judgements would therefore require a body representation incorporating the shape and curvature of the thenar and hypothenar eminences. In contrast, other judgements, such as ones about the line connecting the metacarpophalangeal joint of the thumb and the wrist, might be resolved using a simpler two-dimensional representation of the hand combined with postural feedback.

We found that low amounts of smoothing did not impair participants’ accuracy, but there was a steep decline for more extensive smoothing levels (see coloured lines in figure 5A), suggesting that local geometry at a high level of detail is both perceptually accessible and important for solving the task (see model 3 in figure 5A, bottom row, for the smoothest model where accuracy was above chance level). In contrast, since smoothing did not affect the general outline of the hand model or its overall posture, the accuracy of these judgements did not change (see grey line in figure 5A). Moreover, judgements associated with local three-dimensional geometry were, on average, more accurate than those merely requiring a two-dimensional outline model of the hand (*t*(38) = 2.86, *p* < 0.01, *d* = 0.61, paired *t*‐test, figure 5C), despite the latter both intuitively appearing easier and objectively being located further from the body surface (mean absolute distance 3.8 mm versus 2.8 mm for three-dimensional midpoints, *t*(38) = −7.39, *p* < 0.01, *d* = −1.32, paired *t*‐test). Additionally, a lateralized effect emerged: in experiment 2A, touch points were balanced such that five judgements included the left side of the hand and five the right side (with three focusing on the palm), and comparing their respective accuracy showed significantly higher performance on the right (73%) compared with the left side (59%, t(178) = −3.25, *p* < 0.01, d = −0.48, two-sample *t*‐test). Indeed, mapping all judgements across both experiments onto a standardized reference hand revealed clear spatial clustering: high accuracy on the palm and consistent errors on the wrist, and the sides of the hand (figure 5D). That errors are spatially clustered suggest that they arise from underlying distortions in local body representation rather than random lapses in judgement.

### (e) Boundary judgements are aligned with a typical body model

We selected touch points such that their midpoints were predictably inside or outside the body boundary for most participants. However, as this was not always the case, we three-dimensionally scanned the tested body parts for each participant to measure their individual body shape. We found that for 13 out of 26 total midpoints tested on the hand (across experiments 1A and 2A), some participants’ body shape deviated enough for these points to cross the body boundary from inside to outside or *vice versa*. Similarly, 11 out of 26 midpoints tested on the ankle (across experiments 1B and 2B) spanned both sides of the body boundary across all participants tested. For example, the lateral malleolus typically presents as a pronounced bump on the ankle, causing the line connecting it to the back of the foot to lie outside the body for most participants (see figure 5F, left). However, for a small subset of participants, this bump was much less pronounced and the surrounding region correspondingly almost flat, such that the line fell inside the body instead (see figure 5F, right). Are these individual differences in body geometry reflected in participants’ judgements? In an exploratory analysis, we noticed that participants’ accuracy was often low for judgements in which their individual body shape differed from the typical one (see examples in figure 5E). Indeed, when probing this effect across all data points in the hand baseline (experiments 1A and 2A) and the ankle (experiments 1B and 2B) conditions, we found that perceptual judgements were on average much more accurate in cases when the participant’s body shape agreed with the average shape compared with when the participant’s body differed from the average (figure 5G, typical versus atypical columns). This effect could not be explained by participants with an atypical body exhibiting lower accuracy generally, as their other judgements for different midpoints were much more accurate (figure 5G, control column). In line with these observations, an ANOVA found significant differences between typical, atypical and control judgements (*F*(2,69) = 26.31, *p* < 0.01, *η*² = 0.43) and *post hoc* Tukey tests confirmed that atypical judgements differed from the two other classes (*p* < 0.01 for both), while the accuracy of typical and control judgements did not differ (*p* = 0.65). We note that the difference between typical and atypical body shapes was small, with the body boundary shifting by only 2.1 mm on average between typical and atypical body shapes. Larger discrepancies might well be integrated into people’s body representations. However, on a small scale, when there are individual differences in body shape, participants appear to align their judgements with a more ‘typical’ body rather than their own.

## 3. Discussion

We developed a novel psychophysical paradigm to examine how precisely people judge the boundaries of their own body, specifically whether the midpoint between two brief touches on the skin fell inside or outside the body. Across five experiments, we systematically tested boundary judgements in three conditions: the hand in a relaxed posture (experiments 1A and 2A), the hand in an ‘unnatural’ posture (experiment 2C) and the ankle (experiments 1B and 2B). Despite differing sensory profiles and visual familiarity across these body parts, our findings consistently showed that participants could localize their body boundaries with often striking accuracy; within just a few millimetres of the actual anatomical border, even without visual feedback and when assuming different postures.

### (a) Comparison with previous findings

Previous work on body representations has emphasized large perceptual distortions, which replicate robustly and transfer across different experimental protocols [11]. How can our finding of high perceptual accuracy be reconciled with these prior results? First, the apparent discrepancy might be a consequence of our experimental paradigm. Previous studies, whether on body part size estimation or judgements about tactile distance, specifically asked participants to make size or length estimates. A distorted representation of the hand, for example stretched or compressed along a certain axis, is easily measurable using these paradigms. In contrast, our paradigm assessed whether people are aware of the shape of their body between two touched points. Any systematic distortion (stretch or compression) of these body parts would not affect their overall shape, and perceptual judgements should therefore be unaffected. Thus, if body representations are fine-grained and accurate in their three-dimensional representation but otherwise distorted by appearing elongated or truncated along a certain axis, then our findings are entirely compatible with previous ones. Second, it is possible that we tested body parts where representations are relatively undistorted. The large distortion of the hand representation is mainly evident on the hand dorsum and considerably smaller on the palmar side [20]. While perceptual distortions have been measured on the foot dorsum [21], to our knowledge the ankle has not yet been tested specifically. Additionally, recent findings suggest that observed distortions might be a consequence of cognitive processes rather than the underlying body representation [22]. Our findings demonstrate that local three-dimensional body geometry is accurately represented. Finally, it is possible that our task, specifically asking participants to make judgements about their body boundaries, engaged different representations than those in previous tasks. Given the robustness of previous findings with respect to the specific paradigm used, we believe this to be unlikely.

### (b) Regional differences in body boundary perception

Our results indicated that while the width of the perceived body boundary was larger on the ankle compared with the hand, this difference was not significant and, in any case, smaller than we had anticipated. We predicted a larger discrepancy because the hand possesses denser mechanoreceptor innervation [23], better tactile spatial discrimination and localization [24,25], lower tactile thresholds [26], a disproportionately large cortical representation [27] and higher proprioceptive accuracy [28] compared with the ankle. Moreover, our hands frequently occur within our field of view during manual tasks [29], and the space close to our hands draws attention towards it [30], which is not the case for the foot. Previous studies have noted the importance of vision in shaping body representations [31,32]. Nevertheless, body boundary perception was similar on both body parts, which appear to be represented with high fidelity.

While there was no significant difference between body boundary perception on the hand and the ankle, perceptual accuracy differed spatially across the hand. Specifically, error rates were high on the wrist, even when judging locations that were clearly inside or outside the body. In fact, there appeared to be a sharp edge in accuracy between the hand and the wrist, with the lower palm area judged highly accurately while the areas just proximal to the wrist border were much less so. This pattern of localized errors aligns with prior studies of tactile distance perception, where distortions can cluster around joint boundaries or transitional skin areas, possibly because of segmented body representations ([33,34]; see also [35] for a review). A similar effect might be responsible for our findings. Given that boundary perception was more accurate on the palm compared with the rest of the hand, it is plausible that accuracy could further increase if tested on the fingers or fingertips. These areas are frequently in contact with external objects [36] and are, therefore, potentially more critical for haptic behaviours.

### (c) Fine-grained representation of local body geometry

We found that body boundary perception adapted to changes in posture, meaning that ongoing feedback about the body’s configuration is therefore taken into account when making such judgements. We did not find significant differences in boundary perception between the two postures tested, but previous literature has described postural effects on other measures of body representation [37]. Importantly, postural information alone could not explain the observed results, as coarsening the measured three-dimensional body part models led to considerable decreases in accuracy. In fact, somewhat counterintuitively, we found that participants were significantly more accurate when judgements involved detailed three-dimensional body shape, rather than two-dimensional outlines. These results suggest that participants’ body models incorporate detailed three-dimensional shape information. In the absence of vision, somatosensory feedback might supply some relevant information: skin stretch patterns contain information about three-dimensional body conformation beyond that supplied by proprioceptive feedback [38], and some tactile neurons in the hand have been found to provide ongoing information about the local state of the skin even in the absence of an external force [39]. In line with these findings, anaesthetizing body parts has been found to change perceived body geometry [40–42], suggesting that ongoing feedback is required to maintain these representations and perhaps contributes to them (see also [43] for somatosensory contributions to interoception more broadly). Nevertheless, body models are likely to be constructed and stored, as it is unlikely that all relevant information would be available from sensory feedback [15]. Our finding that in some cases participants’ judgements aligned with a typical rather than their own body would appear to support this statement.

### (d) Boundary perception might reflect typical rather than individual geometry

We found that when a midpoint fell inside or outside the body boundary for most participants, even those whose anatomy diverged from this norm often judged it in line with the majority’s boundary. This suggests that body boundary perception is not determined solely by real-time sensory input, but also by a generalized, internalized model of the body [15,44,45]. One possible explanation for this effect involves statistical learning: over time, individuals observe countless bodies (both their own and others’), gradually internalizing a generalized prototype of where their boundaries should lie. Consequently, when deprived of visual feedback, participants may default to this prototype rather than rely on their unique anatomy. Consistent with this idea, it has been suggested that the way people represent their own bodies is directly influenced by their representations of an average or prototypical body [46], causing perceptual biases that align towards this norm [47,48]. Additionally, previous research has found that people often fail to recognize photographs of their own hands [49,50] and that weaker visual memories of one’s body part correlates with heightened plasticity in its representation [51].

Moreover, individuals rarely direct close visual or tactile attention to their own ankles, which typically lie outside the visual field [52,53]. Over time, people might have observed others’ ankles more frequently and from more diverse perspectives than their own. This scarcity of direct sensory experience specific to one’s own ankle may have amplified reliance on a generalized model of ankle shape and boundaries in our paradigm. Future research should directly test whether body parts with more or less first-person sensory exposure cause measurable body boundary differences. Together, these findings suggest that higher-order body models can override immediate sensory cues and are not fully personalized, perhaps explaining why participants’ judgements often converged towards a generalized boundary prototype.

Yet, the fact that convergence occasionally produced anatomically incorrect judgements raises an important caveat. If a given midpoint (e.g. near the wrist) was truly outside for everyone, an average boundary model would not consistently mislabel it as inside. That it did implies an additional mechanism beyond mere statistical learning or body-part knowledge is responsible for shaping body boundary representations. We observed that, despite strong overall performance, certain midpoints that were persistently misidentified as either ‘inside’ or ‘outside’ when they were clearly the opposite were clustered on the left side of the right hand and around the wrist. These errors persisted even when participants changed hand positions, implying that enhanced proprioceptive cues could not correct them. Nor could a universal average hand boundary explain why midpoints far beyond or within a plausible anatomical limit were misjudged; virtually everyone’s boundary would place these points as such. Instead, the consistency of these errors suggests a distinct, localized distortion or error in the internal mapping of the hand or arm. The fact that such systematic errors were not observed on the ankle might partially be explained by the many more degrees of freedom of movement in the hand and wrist, which, as demonstrated in the manuscript, can frequently cause points to move across to the other side of the body surface. In contrast, the ankle displays fewer degrees of movement, and its range of motion is smaller, such as to preserve local geometry.

### (e) Conclusions and future directions

Taken together, our findings support the notion that the subjective experience of one’s body arises from an integration of immediate sensory input and higher-order, abstract body representations. However, important questions remain regarding the precise balance between these influences. Future research should aim to disentangle the contributions of bottom-up sensory cues and top-down cognitive models in shaping body boundary judgements. Investigating a wider range of body regions, such as the neck, elbow and other joints, could help determine whether the perceptual patterns we observed generalize across the body or vary systematically according to anatomical or functional properties.

The face represents a particularly promising target, given its highly individualized anatomical structure and frequent visual exposure. Knowledge and representation of one’s own face are likely more detailed and distinct from others’, which may reduce reliance on prototypical information. Similarly, research involving individuals with body dysmorphia and related disorders could provide insight into how distorted top-down influences affect body boundary perception (see e.g. [54]), and whether such effects are localized to specific body parts. Finally, the consensus around anatomically implausible boundary judgements appears to operate independently of top-down influences and warrants further investigation. These rare but systematic errors might reflect localized distortions in body representation and highlight the need for finer-grained mapping of perceptual accuracy across the body.

In summary, we found that detailed three-dimensional body geometry is perceptually accessible, with high accuracy both on the palmar surface of the hand and on the ankle. Perceptual judgements of the body boundary adapted to changes in posture, maintaining accuracy and demonstrating the integration of ongoing sensory feedback. Judgements requiring knowledge of detailed, local geometry displayed the highest accuracy and detailed local geometry is therefore represented, though this internal model might partially rely on general knowledge of the shape of the typical body, rather than the participants’ own.

## 4. Material and methods

### (a) Participants

Twenty-one individuals (12 female, 9 male) between the ages of 18 and 27 years old participated in study 1. All participants were right-handed, except for one who was ambidextrous. Study 1 was composed of two experiments: psychophysical testing on the hand (experiment 1A), followed by the ankle (experiment 1B). All 21 participants completed both experiments. Eighteen participants (13 female, 5 male) aged 18 to 28 years old participated in study 2. All but one participant was right-handed. Study 2 included three experiments. The first experiment (experiment 2A), testing the hand using new touch points, was completed by all participants. Seven of them subsequently completed the second experiment (experiment 2B), which included points on the ankle, while the remaining 11 participants completed the third experiment, which tested the hand again, but in a different posture (experiment 2C). See table 1 for assignments of participants to experiments. All participants provided written informed consent prior to the start of data collection. The study protocol was approved by the ethical review board of the School of Psychology at the University of Sheffield (protocol number 060858).

**Table 1.** Distribution of participants across experiments. Both studies included two tasks each.

	task 1	task 2
study 1	experiment 1A—hand relaxed (*n* = 21)	experiment 1B—ankle (*n* = 21)
study 2	experiment 2A—hand relaxed (*n* = 18)	experiment 2B—ankle (*n* = 7)
		experiment 2C—hand non-relaxed (*n* = 11)

### (b) Selection of touch points, midpoints and postures

#### (i) Experiments 1A and 1B

To ensure uniform coverage of perceptual midpoints across participants, predefined locations were selected on the hand and ankle. The chosen points were selected to maximize variation in midpoint locations, ensuring that midpoints were either robustly and clearly falling inside the body, outside or very close to the surface. For the hand (experiment 1A), participants were instructed to rest their right hand in a relaxed posture on the table. Eleven touch points (labelled A–K) were identified on the palmar surface of the hand and the wrist area. Thirteen unique pairs of points were selected to generate midpoints spanning the inside, outside and boundary of the hand. For the ankle, participants were seated in a chair, resting their right foot on the ground at a roughly right angle. Seven touch points (labelled A–G) around the lateral malleolus were identified and then 13 pairs were chosen such that their midpoints again spanned the inside and outside of the body in a range similar to that of the hand (figure 3A,B, top row).

#### (ii) Experiments 2A, 2B and 2C

In experiment 2A, the hand was in the same posture as in experiment 1A. For experiment 2C, participants adopted a new posture, characterized by ulnar deviation at the wrist and partial flexion of the thumb (see figure 4A). This posture was chosen to maximize changes in the distance of the midpoints to the body surface between the two postures. The same set of touchpoints and midpoints were tested in experiments 2A and 2C. For these experiments, a new set of 13 points (labelled A–K) was chosen, with some points identical to those in 1A and others newly chosen. In addition to replicating the results of experiment 1A with new midpoints, we also wanted to specifically force perceptual judgements to be either about boundary depth (i.e. local three-dimensional geometry) or two-dimensional outlines (e.g. edge judgements). The hand was chosen for the posture experiment, because of its high mobility with many degrees of freedom. For experiment 2B, we focused on the ankle again but chose touch points further away from the lateral malleolus, testing the foot outline around the ankle more broadly, rather than detailed local geometry. A set of nine touch points (labelled A–I) was chosen, from which again 13 pairs were selected (figure 3A,B, bottom row).

Sample sizes were chosen based on previous studies in the field examining the accuracy of body representations across multiple sites. Comparing the perceived length or volume of different body parts yielded moderate to very large effects for body parts with the largest differences [14], and we similarly expected a large difference in body boundary perception between the hand and the ankle. Our analysis including 28 participants (across experiments 2A and 2B and the corresponding data from experiments 1A and 2A) was sensitive enough to detect effect sizes of 0.5 and larger at 80% power. Our comparison between different hand postures was mainly intended to confirm that body perception takes into account the hand’s current posture, by testing whether perceptual shifts aligned with physical shifts in the judged midpoints. Given the low number of participants in this task, only effect sizes larger than 0.8 can be reliably detected, and it is therefore possible that body boundary accuracy differs on a smaller scale between postures.

### (c) Experimental procedure

Participants were blindfolded throughout the experiment to eliminate visual feedback. A skin-safe marker pen was used to label the predefined touch points on the skin. After labelling, the participant assumed the tested posture, for which they received gentle guidance by the experimenter, if needed. The relevant body part (hand or ankle) was then scanned in three dimensions. Scanning was performed using a POP 3 Plus (Revopoint 3D, Shenzhen, China) handheld three-dimensional scanner and the accompanying RevoScan software, which generated three-dimensional models. Scanning took a couple of minutes, after which the psychophysical experiment started.

For each individual trial, participants received brief simultaneous touches at two predefined touch points using metal probes with rounded tips roughly 2 mm in diameter. Touches were light and consisted of brief taps on the skin. These were performed by the same experimenter for all participants. Participants were required to make a two-alternative forced choice judgement on whether the perceived midpoint between the two touches fell inside or outside of their body boundary, which was noted by an experimenter. Each experiment included 13 pairs of touch points, which were tested 7 times each, for a total of 91 trials per experiment. The order of stimuli was randomized to prevent response bias and order effects. In the first trial, participants were instructed to take as much time as needed to respond. For subsequent trials, participants had up to 3 s to provide a response. Participants were allowed to take breaks or to relax their hands for a few seconds in between trials, but the majority completed the full protocol in one go. If a pause was taken, participants were carefully guided by the experimenter to assume the original posture. In study 1, participants first completed trials on the hand before moving on to the ankle. In study 2, participants first completed trials in the relaxed hand posture, before moving on to either the ankle (first 7 participants) or the second hand posture (remaining 11 participants).

### (d) Analysis

#### (i) Three-dimensional model processing

Using custom code based on the *pyvista* Python module, touch points were manually identified from the pen markings on the obtained three-dimensional models. Midpoints and their distances from the skin surface were then calculated automatically and exported as csv files. For example, for the hand (experiments 1A and 2A), the average absolute distance from the skin surface across all trials and participants was 3.5 mm (5th percentile: 0.3 mm, 95th: 9.2 mm). As a simple measure of skin curvature, we can also calculate the angle between the two lines connecting the two touch points with the projected midpoint onto the skin. This angle deviated from a flat line by, on average, 12.9° (5th percentile: 1.4°, 95th: 31.7°). In our data, absolute distances and angles were highly correlated (*r* = 0.90) and both metrics therefore yielded equivalent results.

#### (ii) Psychophysical curve fitting

Psychophysical curves were fitted to the data using the *psignifit* Matlab package [55]. Cumulative Gaussian sigmoids were chosen as psychophysical functions together with a single guess rate, as there was no reason to assume any difference in error rates between inside and outside choices. For group-level psychophysical curves, we first averaged distances and perceptual judgements across participants for each midpoint, before fitting a single psychophysical curve. Thresholds at 25%, 50% and 75% were calculated from the psychophysical curves. The 50% threshold was taken as the bias. Width of the body boundary was defined as the physical distance between the 25% and 75% thresholds. Measures of fit differed across participants, but all obtained data were included in all further analyses.

#### (iii) Smoothing analysis

To test whether detailed three-dimensional body shape was taken into account by participants, we calculated whether their judgements were accurate with respect to a series of progressively smoothed body part models. For smoothing, we used the *smoothSurfaceMesh* function, part of the Lidar Toolbox for Matlab 2024a (Mathworks, Natick, MA, USA), which implements a simple averaging filter that iteratively computes a local weighted mean average on a given mesh. This function was run for 50, 500, 2500 and 5000 steps to yield smoothed models 2−5, respectively, with model 1 being the original unsmoothed model. This analysis was run for all hand models in the relaxed posture (experiments 1A and 2A, *n* = 39). We then recalculated the location of each midpoint and its distance to the surface for each of the smoothed models. Finally, we checked whether participants’ responses were correct with respect to these new distances. This analysis was run for each individual participant separately, before averaging.

#### (iv) Comparing accuracy for typical and atypical body shapes

To test whether participants whose body shape differed from the typical body aligned their judgements with their own body or the average, we first determined all midpoints which fell on different sides of the body boundary for at least one participant across all data from the hand in the relaxed posture (experiments 1A and 2A, *n* = 39) and the ankle (experiments 1B and 2B, *n* = 28). For each of these, we then calculated the average distance and took the side of the body boundary on which it fell (inside or outside) as the ‘typical’ body shape. We then calculated the average accuracy of all participants who agreed with this average (typical) and the average accuracy of all who disagreed (atypical). If participants with an atypical body exhibit high accuracy, this indicates that they are judging their own body; conversely, if they display low accuracy, their internal body representation might agree more with the typical shape. For each midpoint and atypical participant, we also calculated their accuracy on all other midpoints (control) to test whether these participants generally show worse performance, independent of any anatomical differences in body shape.

## Data Availability

Anonymized participant data (psychophysical judgements and three-dimensional scans) as well as basic analysis code has been uploaded to Zenodo at [56].

## References

[1] Ihde D. 1973 Sense and significance. Pittsburgh, PA: Duquesne University Press.

[2] Ratcliffe M. 2013 Touch and the sense of reality. In The hand, an organ of the mind: what the manual tells the mental (ed Z Radman). Cambridge, MA: MIT Press.

[3] Ataria Y. 2014 Where do we end and where does the world begin? The case of insight meditation. Philos. Psychol. **28**, 1128–1146. (10.1080/09515089.2014.969801)

[4] Ataria Y, Dor-Ziderman Y, Berkovich-Ohana A. 2015 How does it feel to lack a sense of boundaries? A case study of a long-term mindfulness meditator. Conscious. Cogn. **37**, 133–147. (10.1016/j.concog.2015.09.002) 26379087

[5] Dambrun M. 2016 When the dissolution of perceived body boundaries elicits happiness: the effect of selflessness induced by a body scan meditation. Conscious. Cogn. **46**, 89–98. (10.1016/j.concog.2016.09.013) 27684609

[6] Gallagher S. 2006 How the body shapes the mind. Oxford, UK: Clarendon Press.

[7] de Vignemont F. 2010 Body schema and body image—Pros and cons. Neuropsychologia **48**, 669–680. (10.1016/j.neuropsychologia.2009.09.022) 19786038

[8] Medina J, Coslett HB. 2010 From maps to form to space: touch and the body schema. Neuropsychologia **48**, 645–654. (10.1016/j.neuropsychologia.2009.08.017) 19699214 PMC2813960

[9] Riva G. 2018 The neuroscience of body memory: from the self through the space to the others. Cortex **104**, 241–260. (10.1016/j.cortex.2017.07.013) 28826604

[10] Longo MR, Azañón E, Haggard P. 2010 More than skin deep: body representation beyond primary somatosensory cortex. Neuropsychologia **48**, 655–668. (10.1016/j.neuropsychologia.2009.08.022) 19720070

[11] Longo MR. 2022 Distortion of mental body representations. Trends Cogn. Sci. **26**, 241–254. (10.1016/j.tics.2021.11.005) 34952785

[12] Linkenauger SA, Wong HY, Geuss M, Stefanucci JK, McCulloch KC, Bülthoff HH, Mohler BJ, Proffitt DR. 2015 The perceptual homunculus: the perception of the relative proportions of the human body. J. Exp. Psychol. **144**, 103–113. (10.1037/xge0000028) 25494548

[13] Stone KD, Keizer A, Dijkerman HC. 2018 The influence of vision, touch, and proprioception on body representation of the lower limbs. Acta Psychol. **185**, 22–32. (10.1016/j.actpsy.2018.01.007) 29407242

[14] Sadibolova R, Ferrè ER, Linkenauger SA, Longo MR. 2019 Distortions of perceived volume and length of body parts. Cortex **111**, 74–86. (10.1016/j.cortex.2018.10.016) 30471452

[15] Longo MR, Haggard P. 2010 An implicit body representation underlying human position sense. Proc. Natl Acad. Sci. USA **107**, 11727–11732. (10.1073/pnas.1003483107) 20547858 PMC2900654

[16] Mora L, Cowie D, Banissy MJ, Cocchini G. 2018 My true face: unmasking one’s own face representation. Acta Psychol. **191**, 63–68. (10.1016/j.actpsy.2018.08.014) 30219412

[17] Myga KA, Ambroziak KB, Tamè L, Farnè A, Longo MR. 2021 Whole-hand perceptual maps of joint location. Exp. Brain Res. **239**, 1235–1246. (10.1007/s00221-021-06043-6) 33590275

[18] Longo MR, Haggard P. 2011 Weber’s illusion and body shape: anisotropy of tactile size perception on the hand. J. Exp. Psychol. **37**, 720–726. (10.1037/a0021921) 21480744

[19] Miller LE, Longo MR, Saygin AP. 2016 Mental body representations retain homuncular shape distortions: evidence from Weber’s illusion. Conscious. Cogn. **40**, 17–25. (10.1016/j.concog.2015.12.008) 26741857

[20] Longo MR, Haggard P. 2012 A 2.5-D representation of the human hand. J. Exp. Psychol. **38**, 9–13. (10.1037/a0025428) 21895388

[21] Manser-Smith K, Tamè L, Longo MR. 2021 Tactile distance anisotropy on the feet. Atten. Percept. Psychophys. **83**, 3227–3239. (10.3758/s13414-021-02339-5) 34240341

[22] Peviani VC, Miller LE, Medendorp WP. 2024 Biases in hand perception are driven by somatosensory computations, not a distorted hand model. Curr. Biol. **34**, 2238–2246. (10.1016/j.cub.2024.04.010) 38718799

[23] Corniani G, Saal HP. 2020 Tactile innervation densities across the whole body. J. Neurophysiol. **124**, 1229–1240. (10.1152/jn.00313.2020) 32965159

[24] Mancini F, Bauleo A, Cole J, Lui F, Porro CA, Haggard P, Iannetti GD. 2014 Whole‐body mapping of spatial acuity for pain and touch. Ann. Neurol. **75**, 917–924. (10.1002/ana.24179) 24816757 PMC4143958

[25] Weinstein S. 1968 Intensive and extensive aspects of tactile sensitivity as a function of body part, sex, and laterality. In The skin senses (ed DR Kenshalo), pp. 195–222. Springfield, IL: Charles C. Thomas.

[26] Hennig EM, Sterzing T. 2009 Sensitivity mapping of the human foot: thresholds at 30 skin locations. Foot Ankle Int. **30**, 986–991. (10.3113/FAI.2009.0986) 19796593

[27] Penfield W, Rasmussen T. 1950 The cerebral cortex of man; a clinical study of localization of function. vol. 248. Oxford, UK: Macmillan.

[28] Han J, Anson J, Waddington G, Adams R. 2013 Proprioceptive performance of bilateral upper and lower limb joints: side-general and site-specific effects. Exp. Brain Res. **226**, 313–323. (10.1007/s00221-013-3437-0) 23423167 PMC3627017

[29] Mineiro J, Buckingham G. 2023 O hand, where art thou? Mapping hand location across the visual field during common activities. Exp. Brain Res. **241**, 1227–1239. (10.1007/s00221-023-06597-7) 36961553 PMC10130124

[30] Reed CL, Grubb JD, Steele C. 2006 Hands up: attentional prioritization of space near the hand. J. Exp. Psychol. Hum. Percept. Perform. **32**, 166–177. (10.1037/0096-1523.32.1.166) 16478334

[31] Rakesh Kottu S, Lazar L. 2025 Lack of visual experience leads to severe distortions in the hand representation of the body model. Cortex **183**, 38–52. (10.1016/j.cortex.2024.09.015) 39612568

[32] Shahzad I, Occelli V, Giraudet E, Azañón E, Longo MR, Mouraux A, Collignon O. 2025 How visual experience shapes body representation. Cognition **254**, 105980. (10.1016/j.cognition.2024.105980) 39418855

[33] de Vignemont F, Majid A, Jola C, Haggard P. 2009 Segmenting the body into parts: evidence from biases in tactile perception. Q. J. Exp. Psychol. **62**, 500–512. (10.1080/17470210802000802) 18609376

[34] Knight FLC, Longo MR, Bremner AJ. 2014 Categorical perception of tactile distance. Cognition **131**, 254–262. (10.1016/j.cognition.2014.01.005) 24561189

[35] Tamè L, Longo MR. 2023 Emerging principles in functional representations of touch. Nat. Rev. Psychol. **2**, 1–13. (10.1038/s44159-023-00197-6)

[36] Gonzalez F, Gosselin F, Bachta W. 2014 Analysis of hand contact areas and interaction capabilities during manipulation and exploration. IEEE Trans. Haptics **1412**, 415–429. (10.1109/TOH.2014.2321395) 25532147

[37] Longo MR. 2015 Posture modulates implicit hand maps. Conscious. Cogn. **36**, 96–102. (10.1016/j.concog.2015.06.009) 26117153

[38] Rupani M, Cleland LD, Saal HP. 2025 Local postural changes elicit extensive and diverse skin stretch around joints, on the trunk and the face. J. R. Soc. Interface **22**, 20240794. (10.1098/rsif.2024.0794) 39965643 PMC11835493

[39] Saal HP, Birznieks I, Johansson RS. 2025 Fingertip viscoelasticity enables human tactile neurons to encode loading history alongside current force. Elife **12**, RP89616. (10.7554/eLife.89616) 40511684 PMC12165688

[40] Inui N, Walsh LD, Taylor JL, Gandevia SC. 2011 Dynamic changes in the perceived posture of the hand during ischaemic anaesthesia of the arm. J. Physiol. **589**, 5775–5784. (10.1113/jphysiol.2011.219949) 21946853 PMC3249049

[41] Melzack R, Bromage PR. 1973 Experimental phantom limbs. Exp. Neurol. **39**, 261–269. (10.1016/0014-4886(73)90228-8) 4702820

[42] Walsh LD, Hoad D, Rothwell JC, Gandevia SC, Haggard P. 2015 Anaesthesia changes perceived finger width but not finger length. Exp. Brain Res. **233**, 1761–1771. (10.1007/s00221-015-4249-1) 25788010

[43] Crucianelli L, Ehrsson HH. 2023 The role of the skin in interoception: a neglected organ? Perspect. Psychol. Sci. **18**, 224–238. (10.1177/17456916221094509) 35969893 PMC9902974

[44] Haggard P, Taylor-Clarke M, Kennett S. 2003 Tactile perception, cortical representation and the bodily self. Curr. Biol. **13**, R170–R173. (10.1016/s0960-9822(03)00115-5) 12620204

[45] Tsakiris M. 2010 My body in the brain: a neurocognitive model of body-ownership. Neuropsychologia **48**, 703–712. (10.1016/j.neuropsychologia.2009.09.034) 19819247

[46] Longo MR. 2017 Body representation and the sense of self. In The subject’s matter: self-consciousness and the body (eds F de Vignemont, AJT Alsmith). Cambridge, MA: MIT Press.

[47] Cornelissen KK, Bester A, Cairns P, Tovée MJ, Cornelissen PL. 2015 The influence of personal BMI on body size estimations and sensitivity to body size change in anorexia spectrum disorders. Body Image **13**, 75–85. (10.1016/j.bodyim.2015.01.001) 25697956

[48] Cornelissen PL, Johns A, Tovée MJ. 2013 Body size over-estimation in women with anorexia nervosa is not qualitatively different from female controls. Body Image **10**, 103–111. (10.1016/j.bodyim.2012.09.003) 23102545

[49] Holmes NP, Spence C, Rossetti Y. 2022 No self-advantage in recognizing photographs of one’s own hand: experimental and meta-analytic evidence. Exp. Brain Res. **240**, 2221–2233. (10.1007/s00221-022-06385-9) 35596072 PMC9458563

[50] Wuillemin D, Richardson B. 1982 On the failure to recognize the back of one’s own hand. Perception **11**, 53–55. (10.1068/p110053) 7133936

[51] O’Dowd A, Newell FN. 2020 The rubber hand illusion is influenced by self-recognition. Neurosci. Lett. **720**, 134756. (10.1016/j.neulet.2020.134756) 31945447

[52] Fuentes CT, Longo MR, Haggard P. 2013 Body image distortions in healthy adults. Acta Psychol. **144**, 344–351. (10.1016/j.actpsy.2013.06.012) 23933684

[53] van Elk M, Forget J, Blanke O. 2013 The effect of limb crossing and limb congruency on multisensory integration in peripersonal space for the upper and lower extremities. Conscious. Cogn. **22**, 545–555. (10.1016/j.concog.2013.02.006) 23579198

[54] Keizer A, Smeets MAM, Dijkerman HC, van den Hout M, Klugkist I, van Elburg A, Postma A. 2011 Tactile body image disturbance in anorexia nervosa. Psychiatry Res. **190**, 115–120. (10.1016/j.psychres.2011.04.031) 21621275

[55] Schütt HH, Harmeling S, Macke JH, Wichmann FA. 2016 Painfree and accurate Bayesian estimation of psychometric functions for (potentially) overdispersed data. Vis. Res. **122**, 105–123. (10.1016/j.visres.2016.02.002) 27013261

[56] Blaise C, Clark H, Saal H. 2025 Can we tell where our bodies end and the world begins? Evidence for precise three-dimensional internal body models [Data set]. Zenodo. (10.5281/zenodo.15162847)40795976

